# The Most Interesting Hospital in the World

**Published:** 1891-11-28

**Authors:** 


### THE MOST INTERESTING HOSPITAL IN THE WORLD.

By Our Special Commissioner.

We have visited nearly every foreign country, we possess the plans of all, or nearly all, the principal medical institutions in the world; we are familiar with very many of those who labour in hospitals, and we possess a knowledge of the methods adopted, the systems employed, and the causes of success or failure in their administration. In the course of the last twenty-five years we have inspected many hundreds of hospitals of various countries, managed under all kinds of systems, and we have come to the conclusion that no hospital is so worthy of a visit as the Johns Hopkins Hospital at Baltimore.

Its conception was remarkable. A citizen of Baltimore, who was wealthy, Johns Hopkins by name, conceived the idea of erecting a hospital in his native city, which should be so constructed and organised that it would constitute the completest establishment of the kind in the whole world. He left a letter to the trustees he entrusted with the duty of carrying out his wishes which was so precise and clear in its terms that it might well serve as a model to other millionaires who desire to appropriate some portion of their th for the lasting benefit of mankind.

Johns Hopkins, in selecting his trustees, showed an ntimate knowledge of the capacity of his fellow-citizens, and we think it but just to state, judging these gentlemen wholly by the results which they have achieved, that from first to last they have displayed remarkable wisdom and foresight, and that it is due to them to report that no body of trustees have ever more faithfully or wisely accomplished their trust or better fulfilled the duties which devolved upon them.

The trustees were fortunate in selecting Dr. Billings as their counsellor and guide, who, of all men, is recognised throughout the world as the greatest authority upon the subjects which he has made his own. His devotion to duty, his vast powers for work, his indomitable perseverance, his methods of getting to the bottom of anything he takes in hand, and of thoroughly familiarising himself with principles, have all combined to secure a hospital building and a hospital organisation at Baltimore which is unsurpassed, and which it is probable never will be surpassed, at least in our generation.

Not only have the trustees been fortunate in securing buildings so replete with every modern appliance, constructed upon the most approved principles, and embracing, as they do, many novelties which will no doubt be copied wherever a new hospital building is to be erected in the future, but they have showed that they possess a knowledge of men, which has enabled them to select for the various officers of the institution gentlemen not only of eminence, but of special energy, knowledge, and zeal. It may, therefore, be said, as will become apparent to any visitor of experience, that the Johns Hopkins Hospital to-day is so constituted and so officered, that from ceiling to basement everything bears the stamp of finish, and impresses the expert with a feeling of thankfulness that it should have been possible to establish anywhere such an object lesson for the instruction of the world as this institution undoubtedly is.

At the present time all the objects of the late Johns Hopkins have not been accomplished, because the medical school buildings have yet to be erected, and the school itself organised; still, the existing provisions provide an accommodation for a goodly number of post-graduates, who may here pursue their studies and researches under the most favourable conditions, and find themselves at the same time in quarters so comfortable and so inexpensive as to suffice to attract young men of parts who have taken their degree from every civilised country.

So far as the work of Dr. Billings is concerned, the trustees in their minute of May 13th, 1890, only express the unanimous opinion of the medical world when they declare that "whatever excellences the institution can claim as an advance in hospital construction is due to the great minuteness of knowledge in medical and sanitary science possessed by Dr. Billings, and his familiarity with hospital relief of suffering, obtained through his Government service in the medical department in the late war; his extensive research in what has been done elsewhere, and his aptitude in pursuing, perfecting, and constructing appliances for the proper nursing and care of disease in public institutions, and the watchful zeal with which he has directed every step from the beginning." All this is just and right, but the trustees are entitled to the thanks of everybody interested in the hospital for the wise discernment they have shown in leaving so much to the discretion of Dr. Billings, and also from the fact that they have so carefully administered the funds placed at their disposal that the whole cost of erecting the necessary buildings has been saved out of the income derived from the original bequest of the late Johns Hopkins.

Many men have a gre[illegible]t dread of the Atlantic Ocean, but be that dread what it [illegible]y, we are bound in justice to declare that, in our opinion, no one who is interested in hospitals can form a correct idea of what it is possible to do to secure the maximum of efficiency until they pay a visit and spend three or four days in making a careful investigation of the Johns Hopkins Hospital—its work, it organisation, its building, and its staff.

*(To be continued.)*

